# Post-operative Day Zero Versus Day One Follow-Up for Uncomplicated Cataract Surgery

**DOI:** 10.7759/cureus.29286

**Published:** 2022-09-18

**Authors:** Vaama Patel, Ryan L Freedman, Shibandri Das, Sabba Mansoor, Harsh Parekh, Faisal Ridha

**Affiliations:** 1 Ophthalmology, Visual and Anatomic Sciences and the Kresge Eye Institute, Wayne State University School of Medicine, Detroit, USA; 2 Ophthalmology, Michigan State University College of Human Medicine, Detroit, USA; 3 Ophthalmology, Wayne State University School of Medicine, Detroit, USA

**Keywords:** follow-up study, same day follow-up, pod1, pod0, intraocular pressure, visual acuity, post-operative complications, post-operative management, cataract

## Abstract

Purpose

To compare the postoperative outcomes and management of uncomplicated cataract surgery seen on postoperative day 0 (POD0) versus postoperative day one (POD1).

Methods

A retrospective cohort study of patients who followed up within 0-14 days of their uncomplicated surgery (current procedural terminology code 66984) from December 2018 to March 2020. Those who had perioperative complications, those who had combined glaucoma filtering surgery as well as other minimally invasive glaucoma surgery (MIGS) procedures, and those who did not complete their first two follow-up visits within 14 days of their surgery were excluded. Visual acuity (VA), intraocular pressure (IOP), post-operative interventions, and complications of the first and second postoperative visits were collected.

Results

Of the 665 participants studied, the mean (standard deviation) age was 68 (11) years old and 60% were female (n=304) with a mean (SD) pre-op logarithm of the minimum angle of resolution (logMAR) VA of 0.715 (0.625). About one-third (32%) of patients were seen on POD0. Compared to POD1, a higher percent of patients with glaucoma were seen POD0 (23% vs 14%; p = 0.008). The mean VA on POD0 was 0.840 (0.653), which was significantly worse than the mean VA of 0.539 (0.599) on POD1 (p<0.0001). There was no significant difference in VA by the second post-op visit. IOP did not significantly differ between POD0 and POD1 groups at the first post-operative visit. The most common changes in the post-operative drop regimen were related to IOP and inflammation control. The rate of interventions did not significantly differ between groups (p>0.1). Patients who received intervention on POD0 were not seen significantly sooner at the next follow-up visit compared to those seen on POD0 without undergoing an intervention. The incidence of an IOP spike greater than 30mmHg on POD0 or POD1 was not significantly different between patients with and without underlying glaucoma (overall p = 0.2020; with glaucoma p= 0.1238; without glaucoma p=0.999). Those with a history of glaucoma were not more likely to receive intervention to lower IOP on POD0 versus those seen on POD1 (p = 0.999).

Conclusion

It can be difficult to evaluate patients the day after their uncomplicated cataract surgery, and it is difficult to predict which patients may have post-operative complications. Our study shows no significant changes in management for patients seen on POD0 compared to POD1. Surgeons can expect significantly better visual acuity on POD1, but otherwise, post-operative outcomes were similar between patients seen on POD0 and those seen on POD1. Surgeons may offer the option of a POD0 visit for patients who underwent uncomplicated cataract surgery.

## Introduction

Uncomplicated cataract surgery is a phacoemulsification cataract surgery with no adverse events during the surgery [1]. There are a wide variety of post-operative care practices for uncomplicated cataract surgery. Frequently, patients are seen the day after their surgery. However, more physicians are beginning to evaluate their patients on the same day as their surgery (POD0), so long as the patient’s surgery was uncomplicated.

The American Academy of Ophthalmology (AAO) preferred practice pattern states that patients should return on post-op day one (POD1) if a complication was encountered during their surgery or if they are at higher risk for encountering a post-operative complication [2]. If the patient underwent an uncomplicated surgery with a low risk for a post-operative complication, AAO acknowledges that the patient can be evaluated in the first 48 hours of surgery [2]. Per the Royal College of Ophthalmology guidelines, patients without glaucoma or other comorbid eye conditions do not require a follow-up visit after their uncomplicated cataract surgery [1]. Indeed, many ophthalmologists in the United Kingdom no longer practice following up with their patients on POD0 or POD1, so long as the patient does not have any concurrent diseases of the eye [1].

However, it is difficult to predict whether a patient will develop early postoperative complications, and many physicians prefer to evaluate their patients within a day of their surgery [3-5]. In addition, physicians may also evaluate their post-operative patients the next day to provide reassurance and reiterate post-operative care and eye drop regimen. Resident physicians may prefer to see patients within 24 hours of their surgery to evaluate their surgery and obtain feedback for improvement [4-6].

In recent years, surgeons offer POD0 appointments to patients who have undergone uncomplicated cataract surgery. These patients are scheduled in the surgeon’s clinic on the same day as their surgery for transportation and convenience of time. Many clinician-scientists have debated the safety and efficacy of post-op day one (POD1) visits [1, 3-17]. Few have compared POD0 visits with POD1 visits [6, 10, 12]. The purpose of this study is to compare the postoperative outcomes and management of uncomplicated cataract surgery seen on POD0 with those seen on POD1. We hypothesize that the outcomes and management of patients seen on POD0 of uncomplicated cataract surgery were non-inferior and essentially equal to the outcomes and management of patients seen on POD1 of uncomplicated cataract surgery.

## Materials and methods

We performed a retrospective analysis of patients who underwent uncomplicated phacoemulsification cataract surgery at the Kresge Eye Institute from December 2018 to March 2020. This study follows the guidelines of the Declaration of Helsinki. The Wayne State University Institutional Review Board (WSU IRB) deemed the study IRB exempt (Protocol #: IRB-19-11-1536). The current procedural terminology (CPT) code 66984 identified uncomplicated cataract surgical cases. We reviewed the operative notes to ensure no complications occurred during these cases. We included patients who had cataract surgery combined with iStent placement, a minor glaucoma procedure. We excluded subjects who had complications during their surgery and those who had a combined glaucoma filtering surgery, as well as other minimally invasive glaucoma surgeries (MIGS) such as OMNI Surgical system, Hydrus, or Kahook dual blade. We also excluded those who did not complete their first two follow-up visits within 14 days of their surgery. If a patient had both eyes undergo cataract surgery during this period, only the first eye was recorded. Our sample included surgeries performed by supervised residents and attending physicians.

All patients underwent phacoemulsification surgery which is standard of care, described in many previous studies, and per AAO’s preferred practice patterns with minor variations in the technique, depending on the provider [1-12]. Those who were evaluated on POD0 were seen three to six hours after their surgery. Post-operative standard drops varied by the surgeon, but all had one non-steroidal anti-inflammatory drugs (NSAID) topical medication, one steroid topical medication, and one antibiotic topical medication. These drops were most commonly applied four times per day in the first 24 hours of post-operative care. Patients with glaucoma who were already on IOP lowering medications were often asked to continue their drops after surgery, per physician discretion. Snellen visual acuity (VA) and intraocular pressure (IOP) was recorded for the pre-operative visit and the first and second post-operative visits. Snellen VA was converted to a logarithm of the minimum angle of resolution (logMAR) to allow for statistical calculations. Changes in post-operative management were recorded from the first post-operative visit. Interventions were completed or prescribed at the surgeon’s discretion. Patients who were found to have elevated IOP at the first follow-up appointment received IOP-reducing topical medications or an anterior chamber (AC) paracentesis. If patients were found to have increased levels of inflammation in the AC, they received their steroid drops at a higher frequency. Patients were prescribed additional medications such as sodium chloride hypertonicity ophthalmic solution, cyclopentolate, or antibiotic ointment for corneal edema, photophobia, or ocular surface irritation, respectively. All patients were checked for wound leakage at the first follow-up visit as a standard of care. The above medical and procedural interventions were all recorded for the study. 

The mean IOP and mean VA on POD0 and POD1 were compared with a two-sided unpaired t-test. Changes in medical postoperative care were compared between patients seen on POD0 and POD1 with a chi-squared analysis. Significance was determined by an error of less than 0.05. Statistical analysis was performed using StatView 5.0 software (SAS Institute Inc.; Landau and Rabe-Hesketh, 1999) and SPSS (IBM Corp. Released 2016. IBM SPSS Statistics for Windows, Version 24.0. Armonk, NY: IBM Corp).

## Results

Of the total 665 patients included in this study, about 60% of our patients were female, and the mean age was 68.2 ± 10.5 years old (Table 1). The mean pre-operative logMAR VA for all patients was 0.715 (± 0.625), which translates to about 20/100 Snellen VA. We found a higher proportion of patients with a diagnosis of glaucoma seen on POD0 compared to those seen on POD1 (23% vs. 14%; p = 0.009). In addition, our glaucoma specialists were more likely to see patients on POD0 (Chi-squared analysis; p < 0.001). Additional baseline demographic data can be seen in Table 1, showing an otherwise approximate equal distribution of patients in POD0 and POD1. 

**Table 1:** Study demographics POD0 (Post-operative day zero, same day follow up after cataract surgery); POD1 (post-operative day one, next day follow up after cataract surgery); SD (Standard Deviation); logMAR (logarithm of the minimum angle of resolution); VA (visual acuity); IOP (intraocular pressure). ** indicates p < 0.05

	Total (N=665)	POD 0 (n=162)	POD 1 (n=503)	P-value
Average Age (SD)	68.2 (10.5)	69.5 (10)	67.8 (10.7)	P = 0.0864
Gender				P = 0.57
Male (%)	259 (39)	60 (37)	199 (40)	
Female (%)	406 (61)	102 (63)	304 (60)	
Eye Laterality				P = 0.28
Right (%)	349 (52)	91 (56)	258 (51)	
Left (%)	316 (48)	71 (44)	245 (49)	
Glaucoma History				P = 0.008**
Glaucoma (%)	108 (16)	37 (23)	71 (14)	
No Glaucoma (%)	557 (84)	125 (77)	432 (86)	
Average logMAR pre-op VA (SD)	0.715 (0.625)	0.683 (0.599)	0.725 (0.633)	P = 0.463
Average pre-op IOP (SD)	15.8 (3.7)	16.2 (4.0)	15.6 (3.6)	P = 0.092
Average number of IOP lowering medication (SD)	0.261 (0.682)	1.7 (0.876)	1.5(0.875)	P = 0.156

**Figure 1:**
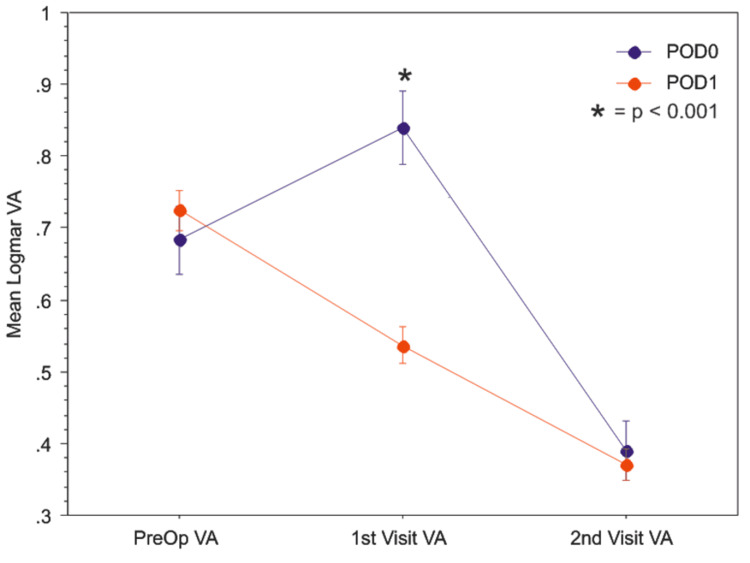
Patients evaluated on the same day as their surgery (POD0, blue) have significantly worse visual acuity (mean logMAR VA ± standard error) compared to those seen the following day (POD1, red) (p<0.001).

Our patients evaluated on POD0 had a mean logMAR VA of 0.840 (Snellen equivalent of about 20/150), which was significantly worse compared to the mean logMAR VA of 0.539 on POD1 (Snellen equivalent of 20/70; p<0.0001) (Figure 1, Table 2).

**Table 2:** Comparison of mean logMAR visual acuity (VA) and mean intraocular pressure (IOP) post-operative care ** indicates p < 0.05

Mean logMAR VA (SD)	Total	POD0	POD1	P-value
Pre-op	0.715 (0.625)	0.683 (0.599)	0.725 (0.633)	0.463
First visit	0.612 (0.625)	0.840 (0.653)	0.539 (0.599)	<0.0001**
Second Visit	0.379 (0.505)	0.390 (0.530)	0.375 (0.497)	0.7424
Mean IOP (SD)				
Pre-op	15.7 (3.7)	16.2 (4.0)	15.6(3.6)	0.092
First Visit	16.4 (5.9)	16.8 (5.7)	16.3 (5.9)	0.3831
Second Visit	15.2 (4.8)	15.6 (5.0)	15.1 (4.7)	0.2018

**Figure 2:**
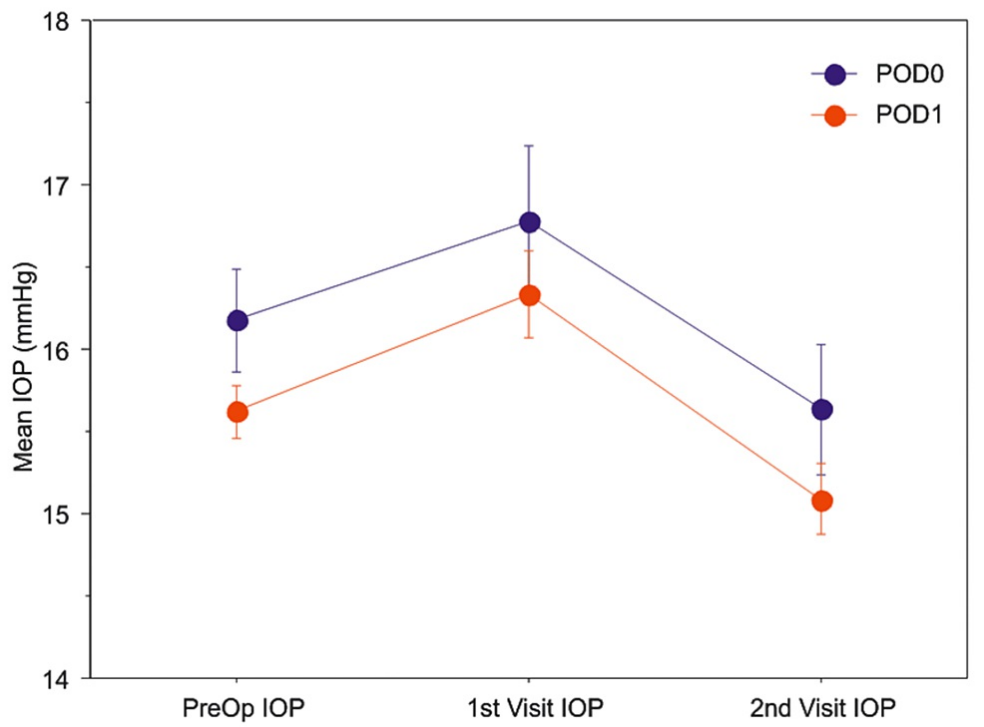
Patients evaluated on the same day as their surgery (POD0, blue) versus the day after their surgery (POD1, red) did not have a significant difference intra-ocular pressure (mean IOP ± standard error) at the first or second post-operative visit.

However, there was no significant difference in the VA on the second post-operative visit occurring between one to two weeks postoperatively. Patients seen on POD0 did not have significantly different mean IOP than those seen on POD1 (Figure 2, Table 2). Patients with glaucoma had a significantly lower mean IOP compared to patients who do not have glaucoma on POD0 (14.6 mmHg vs. 17.4 mmHg; p=0.0079). 

Post-operative outcomes by which a surgeon provided an intervention did not significantly differ between the patients seen on POD0 and the patients seen on POD1 for elevated IOP, increased inflammation, or other conditions such as corneal edema, photophobia, and irritation (Table 3). We did not find any cases of wound leakage at the first follow-up visit identified on either POD0 or POD1.

**Table 3:** Comparison of Interventions during post-operative care

	POD0 (%)	POD1 (%)	P-value
Elevated IOP	10 (6.2)	41 (8.1)	P = 0.4986
Inflammation	4 (2.5)	25 (5)	P = 0.2665
Other (Corneal edema, photophobia, irritation)	5 (3.1)	27 (5.3)	P = 0.2951
Total number of patients who received at least one intervention	18 (11)	77 (15)	P = 0.1899

Those that received intervention on POD0 were not seen significantly sooner for the second visit compared to those seen on POD0 without intervention (p =0.1704). Overall, 23 patients had an IOP spike greater than 30 mmHg, and two patients had an IOP spike greater than 50 mmHg. The incidence of an IOP spike greater than 30mmHg on POD0 or POD1 was not significantly different between patients with and without underlying glaucoma (overall p = 0.2020; with glaucoma p= 0.1238; without glaucoma p=0.999). Those with a history of glaucoma were more likely to have an intervention to lower IOP at the first post-operative visit compared to those without glaucoma (24% vs 12%; p =0.0012). However, those with a history of glaucoma were not more likely to receive intervention to lower IOP on POD0 versus those seen on POD1 (p = 0.999). 

## Discussion

Our study suggests that ophthalmologists may consider POD0 evaluations for uncomplicated cataract surgeries, depending on physician and patient preference as an alternative to seeing patients POD1 for follow-up. There was no significant difference in interventions between patients seen on POD0 compared to patients seen on POD1. Patients with increased levels of inflammation were treated equally on POD0 and POD1. Those with additional interventions for symptoms such as corneal edema, irritation, or photophobia were also equally treated on POD0 and POD1. Although patients with glaucoma were more likely to receive an intervention to lower their IOP, the rate of intervention on POD0 compared to POD1 was not significantly different, as well. About 86% of patients in both groups received standard management, with no postoperative complications identified in our cohort. No cases of wound leakage were identified at the first follow-up visit.

Our posthoc power analysis with a sample size of 665 shows that our chi-square test comparing the overall rates of intervention between the POD0 and POD1 groups achieves 24% power to detect an observed effect size (W) of 0.049 using a 1 degree of freedom chi-square test with a significance level (alpha) of 0.05. This small effect size displays the small magnitude of a difference between the POD0 and POD1 groups, as convention agrees a small effect size is about 0.1, a medium effect size is 0.3, and a large would be 0.5 or larger in a contingency table analysis. As the power is mainly influenced by sample size, effect size, and significance level, the post-hoc power calculation of our study shows a sample size of 665, and a significance level of 80% is reasonable but the effect size of 0.049 is too small. To achieve a medium effect size (W=0.3), the sample size needs 3283 samples, which is unrealistic in our study. So, inversely, we can conclude that there is likely no statistically significant difference between the POD0 and POD1 overall intervention rates with a power of 76%. Although it is possible our conclusion is a type 2 error, it is more likely that there is no significant difference in overall intervention rates between groups, given our large sample size and small effect size. 

On POD0, patients were more likely to have worsened visual acuity compared to their cohorts on POD1. This worsening VA is likely secondary to corneal edema, inflammation, and ocular surface dryness that improved rapidly in the first 48 hours. Elevated IOP was identified in approximately 8% of the initial visits, and our total rate of intervention may have been higher compared to prior studies due to the increased number of resident-led cases in our study. IOP spikes greater than 30mmHg were not more likely to be seen on POD0 compared to POD1. 

Previous studies have suggested eliminating the POD1 visit, recommending the first post-operative evaluation at 1-2 weeks after the patient’s surgery for patients with healthy eyes [3-4, 13-14, 16]. These studies indicated that the main role of the POD1 visit was to offer reassurance, and if an intervention was needed, it was frequently for an elevation in IOP. These studies found that the complications discovered on POD1 were either self-limiting or benign in nature [3, 13-14, 16]. Trufail et al. also suggested that patients can call in to be seen by the ophthalmologist sooner than one to two weeks, should they develop warning symptoms such as pain or worsening visual acuity [3].

Other studies determined that the POD1 visit could not be avoided. These studies determined that if IOP spikes put patients at risk for optic nerve damage, then patients should be seen within the first 24 hours of their surgery. Allan et al. acknowledged that ophthalmologists may hire a non-ophthalmologist to screen the post-operative patient for elevation in IOP, corneal wound leaks, or inflammation in the AC [9]. Cohen et al. and Dinakaran et al. both concluded that the POD1 visit cannot be avoided as the risk of IOP spikes causing damage to the optic nerve is too high [8, 11]. At the time those studies were completed, no definitive recommendations for IOP elevation prophylaxis were available. Since then, randomized clinical trials have found that a single dose of timolol, latanoprost, or travoprost can consistently lower IOP for patients, post-operatively [18]. However, it is still difficult to predict which patients would develop an IOP spike. Some say that IOP spikes were more likely to occur in patients with glaucoma [6]. Our study did find that patients with glaucoma were more likely to receive IOP lowering intervention compared to nonglaucomatous eyes with similar IOP spikes, and the rates of intervention were not significantly different between POD0 and POD1. Providing IOP lowering prophylaxis or intervention for every patient exposes more patients to medication adverse effects needlessly for an event that typically self-resolves [5]. Gupta et al. who studied IOP spikes present in patients with glaucoma after an uncomplicated cataract study stated that an IOP spike greater than 30 mmHg could not be eliminated with prophylactic acetazolamide 250mg. They also recommended evaluating patients on POD1 to receive treatment for an IOP spike, should the patient experience it [15]. Given that our patients with glaucoma were more likely to receive IOP lowering interventions, our surgeons likely had a lower threshold for treating IOP spikes in patients with glaucoma.

Some studies determined that a POD0 visit is non-inferior to a POD1 visit, supporting our conclusions [6, 12]. These studies also agreed on the importance of screening for post-operative IOP elevations. IOP spikes typically peak at 3-8 hours post-operatively [8,10]. Many clinician-scientists believe that these IOP elevations are self-limiting in nature and benign for healthy eyes [5]. Tranos et al. state that so long as patients do not have concurrent glaucoma, patients can be untreated for IOP elevations, and therefore, these patients do not require evaluation on POD0 [12]. Ahmed et al. determined that patients with glaucoma had a significantly higher mean IOP on POD0 compared to patients with no history of glaucoma [10]. Given that the IOP spikes peak on the same day as the operation, we expected a higher intervention rate for lowering IOP spikes on POD0. In spite of this, our study did not find a higher rate of intervention for elevated IOP on POD0, despite having a higher proportion of glaucoma patients seen on POD0. The equal proportion of IOP lowering interventions between the two groups implies that our surgeons did not overtreat nonglaucomatous patients with pressure-lowering medications on POD0. In addition, our patients with glaucoma did have a significantly lower mean IOP compared to patients who do not have glaucoma on POD0. On POD1, patients with glaucoma also did not have a significantly different IOP compared to patients without glaucoma.

Few studies have suggested that significantly increased IOP levels have symptoms such as pain, headaches, or clouded cornea, so patients can call in for a post-operative visit only if they experience these symptoms [6]. Other studies believe that many IOP spikes are asymptomatic or present with vague symptoms [5]. Chong et al emphasized that elevated IOP cannot be determined by slit lamp alone or with symptoms and recommended against omitting a POD1 visit to measure IOP [17]. As such, a visit within the first 24 hours of surgery seems unavoidable. It is not clear whether these elevations in IOP cause clinically significant damage to the eye. Those with glaucoma, on the other hand, were determined to be more likely to be injured with temporary IOP elevations [5, 7, 17]. There is also a rare risk of anterior ischemic optic neuropathy with a significantly elevated IOP, which should not be missed [5]. Our study showed that 3.5% of patients had an IOP greater than 30, and only two patients had an IOP greater than 50. 

Our study had some limitations. First, we had no defined criteria for which patients received the intervention and which patients received standard drops, as this was a retrospective study of multiple surgeons' practices that provided post-operative care in a variety of ways. Instead, we reviewed the post-operative notes to determine whether an intervention was placed. Second, our study had a higher proportion of glaucoma patients in POD0, which may have been a confounding factor in our study. However, our study did not find an association between an elevated postoperative IOP > 30 among patients with glaucoma compared to those without glaucoma, and our results align well with previous studies. Also, IOP lowering interventions were not significantly more likely to occur on POD0, despite those with glaucoma being more likely to receive IOP-lowering intervention compared to those without glaucoma. Therefore, it is unlikely that the higher proportion of glaucoma in the POD0 cohort significantly confounded our results. Previous studies have acknowledged increased patient satisfaction when following up on the following day of their surgery [19]. For future studies, a subjective survey on patient satisfaction with post-operative care on POD0 versus POD1 should be studied.

## Conclusions

The common practice to evaluate a patient the day after their surgery can be difficult for the patient, the surgeon, and the care center for financial and logistical reasons. However, it is difficult to predict which patients will have a post-operative complication for residents in training and attendings alike. Preemptively treating patients with an IOP lowering drop or medication before the surgery is also costly and ineffective, as about 85% of patients commonly do not experience an IOP elevation in the post-op setting. A solution can be to provide the option of either a POD0 or POD1 visit depending on physician and patient preference. Our study shows no clinically or statistically significant changes in management for patients seen on POD0 compared to POD1. Surgeons can expect significantly better visual acuity on POD1, but otherwise, post-operative outcomes were similar between patients seen on POD0 and those seen on POD1. Future studies should examine patient satisfaction on POD0 compared to POD1 evaluations.
